# Dissecting social interaction: dual-fMRI reveals patterns of interpersonal brain-behavior relationships that dissociate among dimensions of social exchange

**DOI:** 10.1093/scan/nsz004

**Published:** 2019-01-15

**Authors:** Beáta Špiláková, Daniel J Shaw, Kristína Czekóová, Milan Brázdil

**Affiliations:** 1 Behavioural and Social Neuroscience Research Group, Central European Institute of Technology, Masaryk University, Kamenice 5, Brno, Czech Republic; 2 Faculty of Medicine, Masaryk University, Kamenice 5, Brno, Czech Republic; 3 Department of Psychology, School of Life and Health Sciences, Aston University, Birmingham, UK

**Keywords:** social interaction, hyperscanning, cooperation, competition, interpersonal brain-behavior dependencies

## Abstract

During social interactions, each individual’s actions are simultaneously a consequence of and an antecedent to their interaction partner’s behavior. Capturing online the brain processes underlying such mutual dependency requires simultaneous measurements of all interactants’ brains during real-world exchange (‘hyperscanning’). This demands a precise characterization of the type of interaction under investigation, however, and analytical techniques capable of capturing interpersonal dependencies. We adapted an interactive task capable of dissociating between two dimensions of interdependent social exchange: goal structure (cooperation *vs* competition) and interaction structure [concurrent (CN) *vs* turn-based]. Performing dual-functional magnetic resonance imaging hyperscanning on pairs of individuals interacting on this task, and modeling brain responses in both interactants as systematic reactions to their partner’s behavior, we investigated interpersonal brain-behavior dependencies (iBBDs) during each dimension. This revealed patterns of iBBDs that differentiated among exchanges; in players supporting the actions of another, greater brain responses to the co-player’s actions were expressed in regions implicated in social cognition, such as the medial prefrontal cortex, precuneus and temporal cortices. Stronger iBBD during CN competitive exchanges was observed in brain systems involved in movement planning and updating, however, such as the supplementary motor area. This demonstrates the potential for hyperscanning to elucidate neural processes underlying different forms of social exchange.

## Introduction

Humans engage in a variety of social exchanges on a daily basis; we interact not only with friends and loved ones, but also with rivals and strangers. Despite our proficiency in negotiating such interactions, each one emerges through a highly complex and dynamic process; even in a simple dyadic exchange, for instance, the actions of each individual are mutually and directly influenced by the prior and present behavior of their interaction partner, and, simultaneously, serve to mutually and directly influence the other’s behavior. In this sense, social interactions between two individuals comprise a ‘two-in-one’ dynamic, whereby the actions of each person are simultaneously a consequence of and antecedent to their partner’s behavior (Koike *et al.*, 2015). Furthermore, each interactant can alternate between different roles during the course of a dyadic exchange. Understanding the brain processes that give rise to such mutual dependency within the non-linear context of social interaction is central to social neuroscience, but this remains somewhat of a ‘dark matter’ (Schilbach *et al.*, 2013).

To investigate the neural processes underlying social behavior, neuroscientists have turned typically to the classic sensory neuroscience approach—individuals’ brain responses are measured while they evaluate experimental social stimuli in isolation. While this has unveiled numerous brain systems underlying social information processing (Van Overwalle, 2009), such a ‘spectator science’ offers little insight into how these systems are modulated during social interaction; by considering individuals as detached observers, we cannot investigate how they respond online to the behavior of our interaction partners (Konvalinka and Roepstorff, 2012; Schilbach, 2014; Hari *et al.*, 2015). Indeed, increasing awareness that social cognition differs fundamentally during real interaction compared to mere observation has led to calls for ‘real-world’, ‘*in situ*’ or ‘two-person’ social neuroscience (Hari *et al.*, 2013; Schilbach *et al.*, 2013; Kasai *et al.*, 2015), whereby the brains of two or more interactants are measured simultaneously while they engage with one another in real-time social exchanges. This ‘hyperscanning’ technique requires a number of methodological and technical developments before it can be used to advance the field of social neuroscience, however, and this was the focus of the present study.

Liu and Pelowski (2014) proposed that three distinct dimensions of interaction must be delineated: the goal structure (competitive *vs* cooperative), interaction structure [concurrent (CN) *vs* turn-based (TB)] and task structure (independent *vs* interdependent). For example, although sporting activities share the common characteristics of a competitive goal, they differ in both interaction and task structures; while opposing tennis players take turns to return a ball, and each individual’s shot is directly dependent upon the prior move of their opponent, individuals in a race compete with one another concurrently and independently. Similarly, although members of a band must cooperate with one another to achieve harmony, they can do so by aligning instrumental outputs simultaneously (CN) or in a sequential (TB) manner, and independently (solo) or interdependently (duet). Since the main benefit of hyperscanning is the ability to explore interaction *in vivo*, it is essential to dissociate among these discrete forms of social exchange (Konvalinka and Roepstorff, 2012). To our knowledge, however, all existing hyperscanning studies have focused on either the goal or task structure—none have explored the interaction structure. In order to understand the neural systems through which the mutual dependency of behavior emerges during social interaction, we must first elucidate the brain processes that are modulated online during these dissociable dimensions of social exchange.

Hyperscanning has undergone a number of technical developments; it has been performed successfully with functional magnetic resonance imaging (fMRI), electroencephalography (EEG), functional near-infrared spectroscopy (fNIRS) and magnetoencephalography (MEG; for reviews, see Scholkmann *et al.*, 2013; Babiloni and Astolfi, 2014). With these techniques, neuroscientists have been able to identify neural processes engaged during various forms of social exchange, from interpersonal motor synchronization (Naeem *et al.*, 2012; Osaka *et al.*, 2014) and joint-action tasks (Funane *et al.*, 2011; Cui *et al.*, 2012) to verbal communication (Jiang *et al.*, 2012; Spiegelhalder *et al.*, 2014) and economic exchanges (King-Casas *et al.*, 2005; Shaw *et al.*, 2018). Further, a range of analytical techniques has been developed to detect mutual dependencies in the brain responses of interacting individuals, from inter-subject correlation (Liu *et al.*, 2015; Koike *et al.*, 2016; Shaw *et al.*, 2018) to sophisticated measures of inter-brain coherence (Babiloni and Astolfi, 2014; Stolk *et al.*, 2014; Liu *et al.*, 2016; Nozawa *et al.*, 2016; Tang *et al.*, 2016; Toppi *et al.*, 2016; for reviews, see Babiloni and Astolfi, 2014; Hasson and Frith, 2016). Although these analytical techniques are capable of measuring symmetrical brain responses between two interacting individuals (‘neural alignment’), this might fail to capture other forms of neural interdependencies during social exchange; while such symmetry might be expected between brains exposed to the exact same stimulus, indicating shared processing or meaning extraction (Hasson and Frith, 2016), interpersonal brain responses are unlikely to take this form during the sequential and non-linear dynamic of naturalistic dyadic exchange. In such contexts, the brain responses of each interactant are likely to reflect a reaction to their partners’ behavior, which might give rise to temporally lawful but ‘asymmetric’ interdependencies. It is therefore necessary to develop and optimize new analytical techniques that are capable of capturing the brain responses of one individual that are modulated by, or dependent upon, the behavior of their interaction partner (Burgess, 2013; Hari *et al.*, 2015), that is, interpersonal brain-behavior dependencies (iBBDs).

In this study, we measured the brain responses of two individuals simultaneously with dual-fMRI while they interacted with one another in a task capable of distinguishing between different dimensions of social interaction, namely, an adaptation of the interactive pattern game (PG; Decety *et al.*, 2004). By measuring both players’ brains simultaneously during this game, we were able to capture neural responses in both interactants’ brains that were modulated online by their co-player’s behavior during an ecologically valid social context, one in which an exchange emerged through the participation of ‘both’ individuals (Schilbach *et al.*, 2013). To investigate brain responses that underlie the mutual dependency characterizing naturalistic dyadic exchange, we considered only the interdependent level of task structure, that is, when the task outcome is dependent upon the performance of both interactants and the performance of each interactant is mutually dependent upon their interaction partner. We then measured iBBDs by modeling the brain responses of one individual as systematic reactions to the other’s behavior (Hasson and Frith, 2016). By separating both interaction and goal structures, we were able to investigate whether iBBDs differed across combinations of CN and TB, cooperative and competitive interactions. Based on the previous research (Krill and Platek, 2012), we predicted strong brain responses to the behavior of an interaction partner in the reward system [e.g. ventral striatum and anterior cingulate cortex (ACC)] during cooperative compared with competitive exchanges. In contrast, we expected stronger iBBDs in brain areas implicated consistently in socio-cognitive capacities (e.g. mentalizing) during competition given the increased need to predict an opponent’s upcoming moves [e.g. medial prefrontal cortex (mFPC); Carlson *et al.*, 2013]. Finally, since there is a higher demand on attention and movement planning during CN relative to TB interpersonal behavior, we hypothesized that there would be greater iBBDs in brain regions associated with attention and movement planning; specifically, the frontoparietal attention network and the supplementary motor area (SMA; Cona and Semenza, 2017). Conversely, in TB exchanges we expected stronger brain responses to an opponent’s behavior in brain areas implicated in behavioral inhibition (e.g. pre-supplementary motor cortex; Nachev *et al.*, 2008) and self-other distinction (e.g., temporoparietal junction and precuneus; Brass *et al.*, 2009; Reniers *et al.*, 2014).

## Methods

### Participants

We recruited 44 individuals (22 males) from Brno, Czech Republic. The mean (s.d.) age of this sample was 22.37 (1.91) years. These participants were paired into same-sex dyads (11 male–male) matched on self-evaluated handedness (40 right-handers), age [mean (s.d.), 6.27 (4.32) months] and education (highest qualification achieved). Importantly, the participants comprising each dyad were unacquainted with each other prior to the day of the experiment; they were introduced to one another for the first time upon their arrival to the scanning facility and instructed together about the task and the scanning procedure. The study was approved by the Research Ethics Committee of Masaryk University, and all participants gave their informed consent prior to the scanning procedure. Participation was rewarded with 200 CZK (~€8).

### The PG

**Fig. 1:**
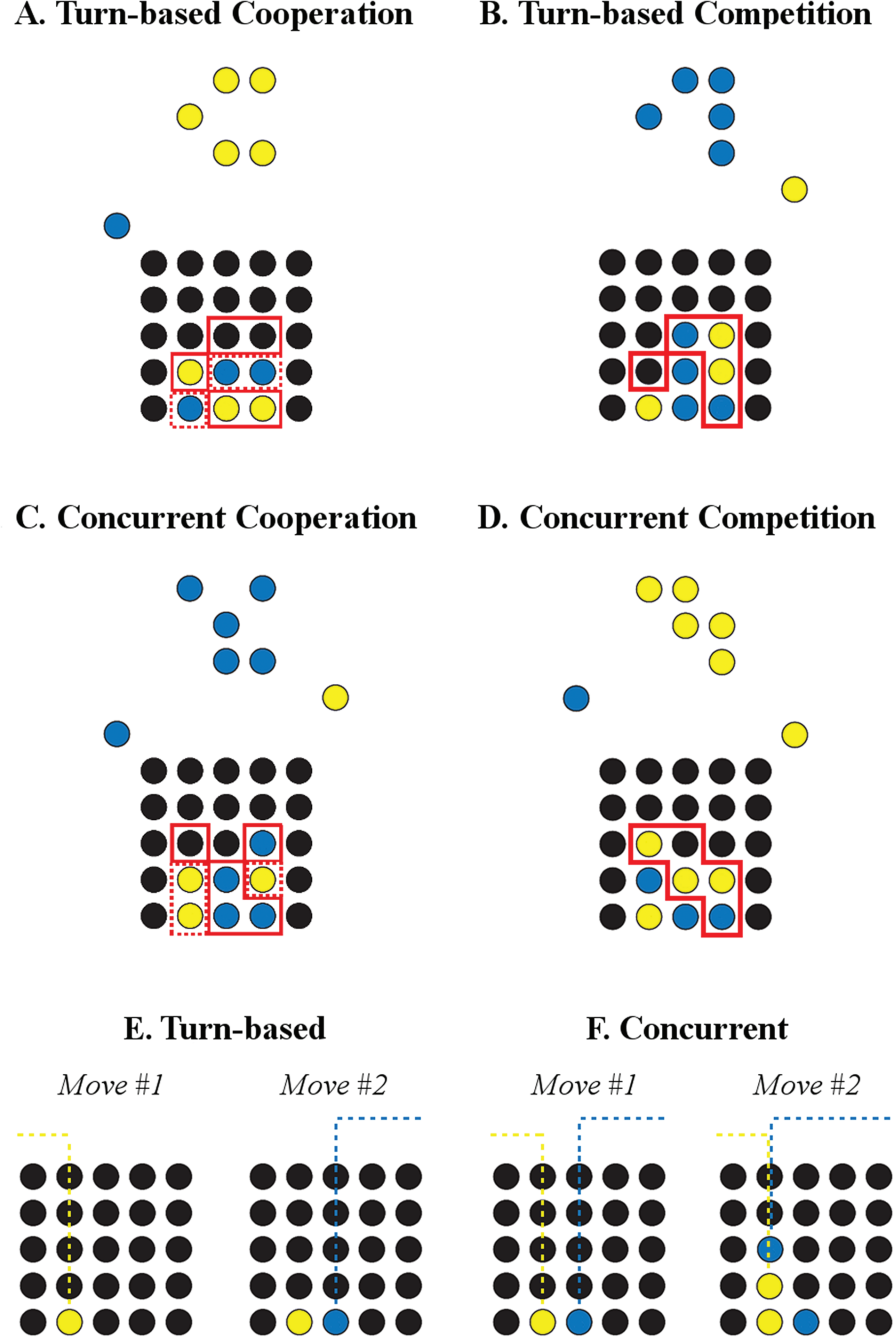
Snapshots of Turn-based (TB) cooperation **(A)** and competition **(B)** rounds and Concurrent (CN) cooperation **(C)** and competition **(D)** rounds and schematics of sequential player moves in (TB) **(E)** and CN **(F)** rounds. In (A–D), the Builder is assigned the same color as the depicted target pattern and scores by placing tokens in locations that recreate the pattern (indicated by solid red lines). The Other scores by placing their tokens in locations that serve to help (dashed red lines) or hinder the Builder; since the latter is achieved by placing tokens within the pattern space, thereby obstructing the Builder, the scoring location of Others and Builders are the same in competitive rounds (solid red lines). In (E), players take turns to move their tokens. In (F), both players can move simultaneously; if both players chose to move their token to the same location then the Builder’s token (lower) is positioned in the first available space, and the Other’s token is positioned above it.

In the PG, two players either cooperated or competed with one another over recursive rounds to reconstruct patterns comprised of blue and yellow tokens (Figure 1). At the beginning of the game, each player was assigned to one color—either blue or yellow—which remains fixed throughout. On any given round, one player was assigned the role of the Builder, whose goal was to recreate the target pattern as closely as possible. Due to the characteristics of the patterns, however, the Builder could never recreate the pattern perfectly on their own. The second (‘Other’) player was instructed to either help the Builder (‘Helper’) or prevent them from reconstructing the pattern (‘Hinderer’), and this instruction defined two experimental conditions: cooperation and competition, respectively. In a control condition, the Other was instructed to simply observe the Builder without contributing any tokens. Participant roles alternated on each round.

Before each round, an instruction was presented for 3 s that allocated each participant to a player role (e.g. ‘Yellow builds, Blue helps’). This was followed immediately by a white fixation cross visible for 1 s, before the round began. Every round began with the players’ tokens presented on either side of the monitor above the playing board (e.g. a blue token on the left side and a yellow token on the right of the monitor; Figure 1). Players then moved their respective token either left or right to the desired columnar location, and then dropped the token into the lowest empty row. In our adaptation, rounds were played iteratively in two separate blocks; in the first block, players took turns sequentially to place their tokens (TB); in the second, participants were free to place their tokens simultaneously (CN). In the TB block, the Builder always placed the first token, immediately after which another token appeared for the Other. In the CN condition, the Builder’s token was always in the lower row, closer to the playing board; as such, if both players attempted to place their token at the same columnar position at the same time, the Builder’s token always dropped to the lowest row and the Other’s token was positioned above it (Figure 1F). In every round of the game, each participant had five tokens to place and every round lasted for a maximum of 25 s. After this time limit, a new round began regardless of how many tokens had been placed. Both the TB and CN blocks consisted of 16 cooperative, 16 competitive and 16 control rounds. These 48 rounds were presented in pseudorandom order, such that no single round type occurred more than three times in a row. It is important to stress that, since all players played an equal number of rounds as Builders and Others, we were able to assess iBBDs during this interactive game in all 44 individuals.

Players moved their tokens via four-button response boxes, on which the buttons were organized horizontally in a single row; the left- and right-most buttons moving tokens one column to the left or right, respectively, and either of the two center buttons caused the token to be dropped into the lowest available row. Before the scanning session, both participants performed four practice control rounds of the PG to familiarize themselves with the task. The entire protocol was coded using MATLAB and Statistics Toolbox (v2016b, The MathWorks, Inc.; RRID:SCR_001622) and the Cogent 2000 toolbox (developed by the Cogent 2000 team at the Functional Imaging Laboratory and the Institute of Cognitive Neuroscience and Cogent Graphics developed by John Romaya at the Laboratory of Neurobiology at the Wellcome Department of Imaging Neuroscience; RRID:SCR_015672).

### MRI data acquisition

Brain images were acquired using two identical 3T Siemens Prisma scanners located in adjacent rooms within the same facility and a 64 channel HeadNeck coil. High resolution T1-weightened structural images were first recorded (MPRAGE; TR/TE, 2300/2.33 ms; flip angle, 8°; matrix, 240 × 224 × 224; 1 mm^3^ voxels). Functional imaging data were then recorded in two sequential runs, each containing 570 volumes (~20 min); the TB block was always followed by the CN block. Blood-oxygen-level dependent (BOLD) images were obtained with T2^*^-weighted echo planar imaging, with parallel acquisition (i-PAT; GRAPPA acceleration factor, 2; axial slices, 34; TR/TE, 2000/35 ms; flip angle, 60°; matrix, 68 × 68 × 34; 3 × 3 × 4 mm voxels). Axial slices were acquired in interleaved order. To ensure the synchronization of the scanners we used an external programmable signal generator to begin the acquisition sequence (Siglent SDG1025; www.siglent.com). Scanners were connected to a stimulation computer via parallel ports, through which radio frequency pulse timings were recorded [asynchrony in volume acquisition: mean (s.d.), 1.69 (0.65) ms].

### Behavioral data

For each round of the PG we recorded all button presses by both players and the final layout of tokens on the playing board. We could then recreate offline the moves of each player in every round. Since TB and CN rounds may have differed in length and, therefore, the number of total moves afforded, we expressed the number of successful placements as a proportion of all moves. For each participant, we calculated the proportion of successful moves they made in both roles under each condition; for Builders, a successful move was defined as any placement that served to partially recreate the target pattern; for Helpers, it was any token placed in a position that provided support to the Builder, while for Hinderers, it was any placement within the desired pattern (thus preventing the Builder from making that same successful placement; Figure 1). For example, in the TB block each participant played eight cooperation rounds in the role of Builder; with five tokens in each round they had the opportunity to make 40 successful placements over the course of the game. Since each participant played the role of Builder and Other on alternating rounds, proportions of successful placements for each player role in each condition were assessed with a 2 (role: Builder *vs* Other) × 2 (goal structure: Cooperate *vs* Compete) × 2 (interaction structure: TB *vs* CN) within-subject analysis of variance.

### Neuroimaging data

Functional and structural brain images were analyzed using the variety of utilities packaged within FMRIB’s software library (FSL; Jenkinson *et al.*, 2012; SCR_002823).

#### Pre-processing

Each of the four functional time-series for a given pair (two players × two blocks of PG rounds) was pre-processed separately; first, motion correction was performed with MCFLIRT (Jenkinson *et al.*, 2002). To remove any residual motion artefacts, or signal caused by physiological noise (e.g. heart rate and respiration), we performed independent component analysis with MELODIC (Beckmann and Smith, 2004) to identify 50 spatial and temporal components of the BOLD signal. Artefactual components were identified automatically using the Spatially Organized Component Klassifikator (Bhaganagarapu *et al.*, 2013), and any signal corresponding to these problematic components was regressed out of the time-series using ‘fsl_regfilt’. Slice-timing correction for interleaved slice acquisition was then applied to these cleaned functional images, and each time series was then high-pass filtered across time (Gaussian-weighted least-squares straight-line fitting; sigma, 50.0 s) and spatially smoothed with a 5 mm full-width half-maximum Gaussian kernel. Using FLIRT, the time series was registered to a corresponding high-resolution structural image using Boundary-based Registration, and this, in turn, was registered linearly to the MNI-152 template (12 degrees of freedom).

#### General linear modeling

**Fig. 2:**
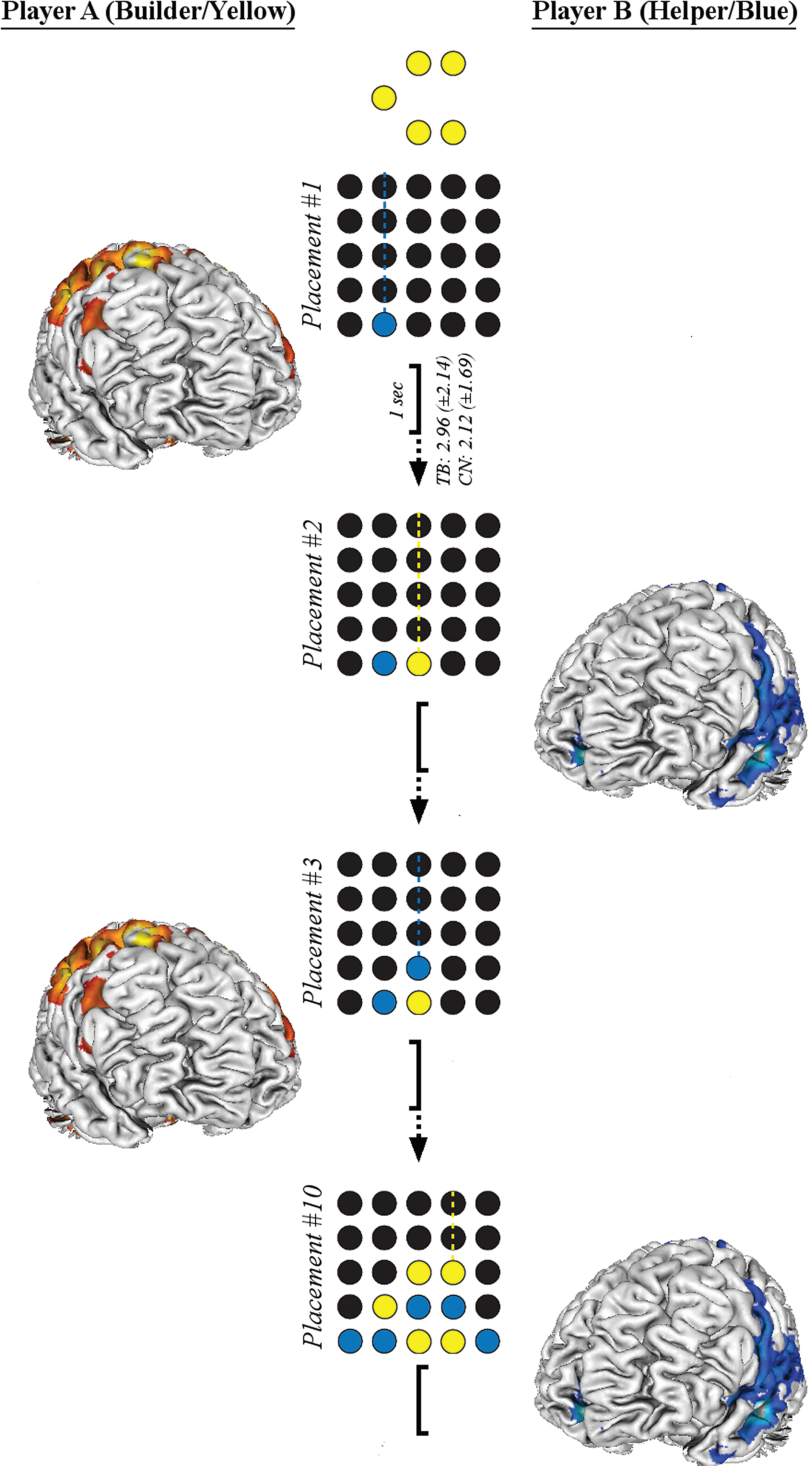
Schematic of the GLM procedure. This illustrates the timings of four token placements during an example TB cooperation round, in which Helper (blue) takes turns with a Builder (yellow) to assist them in recreating the target pattern. The brain responses of each player are modeled in the 1 s period immediately following their co-player’s token placement. The mean (±s.d.) interval between the co-player’s token placement and the individual’s own subsequent placement are shown for both TB and CN rounds. On CN rounds particularly, the individual’s own placement sometimes occurred within this 1 s period, but brain responses specific to an opponent’s token placement and independent of the player’s own moves were identified by subtracting those modeled during a control condition (see text for further details).

With FEAT, general linear modeling (GLM) was used to identify brain signals in each of the 44 participants that were elicited as a direct response to their interaction partner’s prior behavior; specifically, in an event-related fashion we modeled the brain activity of each individual in the 1 s period immediately following each of their partner’s token placement (see Figure 2). In a two-step process, fixed-effect analyses were performed for the following parameter estimations at the individual level: Builders’ responses to the moves of the Other under the cooperation (COO*_Builder_*) or competition condition (COM*_Builder_*); Others’ responses to the moves of Builders under the cooperation (COO*_Other_*) or competition condition (COM*_Other_*); and, in the control condition, the individual’s brain responses while playing the role of Builder and attempting to recreate the pattern without any help or hindrance (CTL*_Builder_*). Importantly, by modeling brain responses recorded during a player’s own token placement in the control condition we were able to distinguish between those reflecting a reaction to their partner’s token placement and those elicited during their own subsequent action (see below). Event-related responses were modeled as stick functions with 1 s duration, convolved with a double-gamma hemodynamic response function. Through combinations and comparisons of these first-level parameters estimates, we then performed group-level whole-brain random-effects analyses with FLAME to examine the main effects of role, goal and interaction; the two-way interactions of role-by-goal, role-by-interaction and goal-by-interaction; and the three-way interaction of role-by-goal-by-interaction (see Supplementary data for full contrast specifications). Since non-parametric permutation inference offers more precise control over false positives than other methods of multiple comparison correction (Eklund *et al.*, 2016), group-level statistical maps were corrected across space using ‘randomize’ (Winkler *et al.*, 2014) with 5000 permutations and threshold-free cluster enhancement (Smith and Nichols, 2009).

## Results

### Behavior

**Fig. 3:**
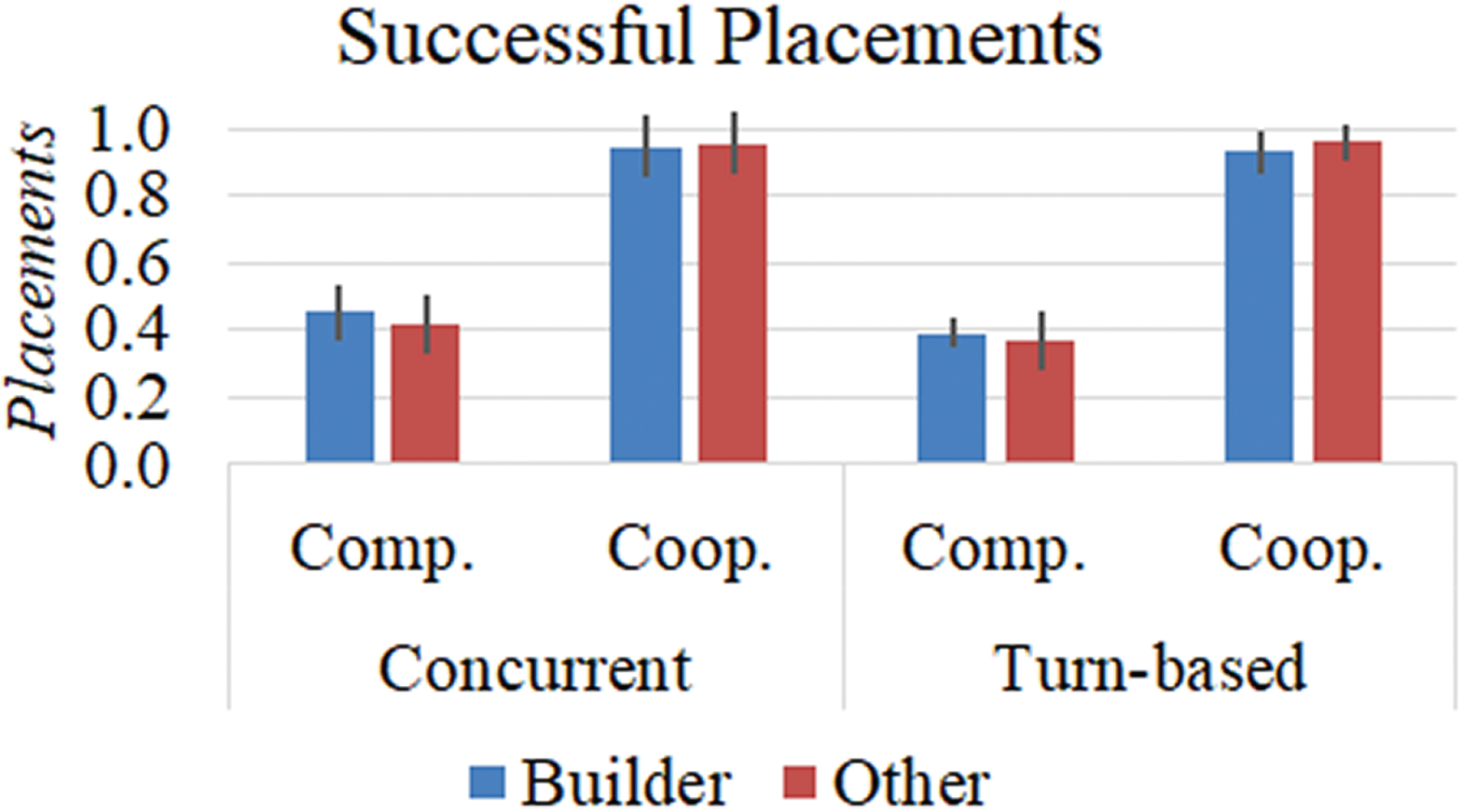
Behavioral data. Mean (±SEM) proportions of successful token placements achieved by Builders (blue) and Others (red) in both levels of goal and interaction structures.

First, for each participant we computed the proportion of all moves that served as successful token placements in the Builder or Other role. There was no main effect of role (F[1,43] = 0.73; *P* = 0.40; ${\eta_{p}^{2}}$ = 0.13), but a main effect of goal confirmed that players made more successful placements in both roles under the cooperation relative to the competition condition (0.95 [±0.01] *vs* 0.41 [±0.01]; F[1,43] = 3655.31; *P* < 0.001; ${\eta_{p}^{2}}$ = 1.00). A main effect of interaction structure demonstrated a higher proportion of successful placements on CN compared with TB rounds (0.69 [±0.01] *vs* 0.66 [±0.01]; F[1,43] = 7.36; *P* = 0.010; ${\eta_{p}^{2}}$ = 0.76). A role-by-goal interaction (F[1,43] = 14.23; *P* < 0.01; ${\eta_{p}^{2}}$ = 0.96) revealed an increased success rate for Helpers compared with Builders in the cooperation condition (0.96 [±0.01] *vs* 0.94 [±0.01]) but greater success for Builders relative to Hinderers in the competition condition (0.39 [±0.01] *vs* 0.42 [±0.01]). There was no interactive effect of a role-by-interaction (F[1,43] = 1.66; *P* = 0.205; ${\eta_{p}^{2}}$ = 0.04). Further, a significant goal-by-interaction effect (F[1,43] = 12.09; *P* = 0.001; ${\eta_{p}^{2}}$ = 0.93) revealed that, while the increased proportion of successful placements made during CN relative to TB rounds was significant under the competition condition (0.43 [±0.01] *vs* 0.38 [±0.01]; *P* < 0.01), this was not the case under the cooperation condition (0.95 [±0.01] *vs* 0.95 [±0.01]; *P* = 0.63). We also found no interactive role-by-goal-by-interaction effect (F[1,43] = 0.10; *P* = 0.756; ${\eta_{p}^{2}}$ = 0.06). These results are illustrated in Figure 3.

### GLM results

**Fig. 4:**
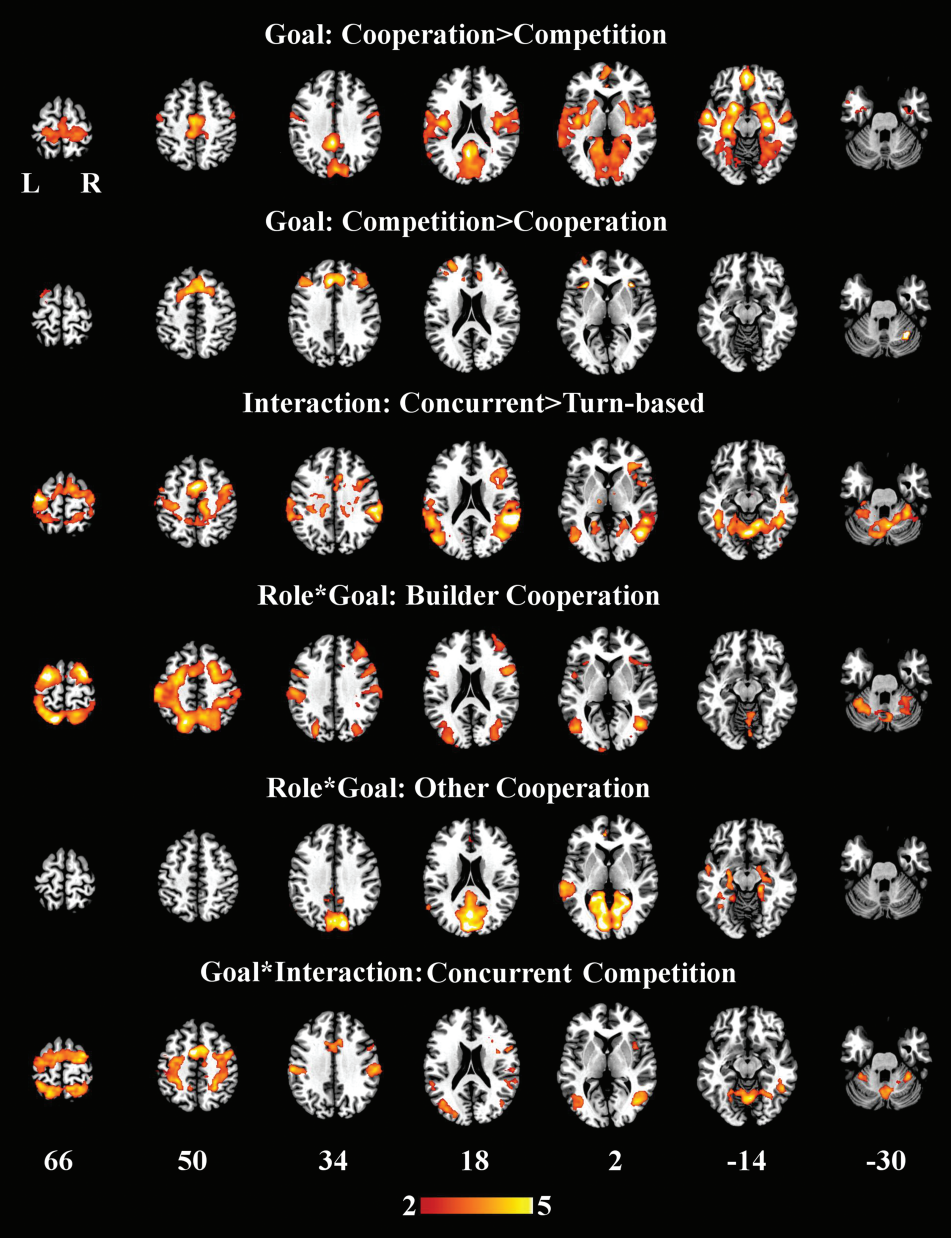
Neuroimaging data from group analyses. Rows present selected axial slices illustrating t-maps in which clusters of BOLD response expressed differential iBBDs among conditions after thresholding (*P*< 0.05) with non-parametric permutation bootstrapping. Note: t-maps are overlaid onto the Colin27 template (Holmes *et al.*, 1998). Values at the bottom of the image present t-coordinates of corresponding axial slices, in MNI space.

Interpersonal brain-behaviour dependency (iBBD) was measured by modeling the brain responses of one individual in the 1 s period following the preceding token placement of their co-player. The mean (±s.d.) duration between a co-player’s preceding token placement and the player’s own subsequent move was 2.96 (±2.14) s in the TB conditions and 2.12 (±1.69) s in the TB conditions. Importantly, by subtracting brain responses measured during the control condition from those recorded in the experimental conditions, we were able to identify the brain responses reflecting reactions to a co-partner’s token placements independently of those elicited during a player’s own moves. Localized brain signals reflecting these neural responses to a co-players’s moves (iBBDs) expressing the contrasts between different dimensions of interaction are detailed in Tables 1–3 and illustrated in Figure 4. Clusters expressing each contrast were identified according to FSL’s ‘cluster’ utility.

**Table 1:** Clusters iBBDs expressing the main effect of goal

Cooperation > competition									Competition > cooperation							
Cluster	Voxels	Label		Max	x	y	z		Cluster	Voxels	Label		Max	x	y	z
3	36910	Putamen	L	8.07	−18	8	−8		5	3770	dmPFC	L	7.49	−4	24	42
			R	7.1	20	14	−6					R	6.48	4	26	44
		MTG	L	7.4	−56	−6	−14						6.89	10	24	44
		Hippocampus	R	7.38	30	−18	−12						6.55	10	32	40
		Precuneus	L	6.87	−4	−58	20				SMA	L	6.48	−8	16	46
		Rolandic operculum	L	6.59	−40	−14	16				MCC	L	7.03	−4	28	36
2	1492	vmPFC	L	6.67	0	42	−10		4	1165	MFG	L	5.54	−30	54	10
				6.58	−2	50	−12						5.21	−46	34	28
				6.4	0	56	−8						5.09	−44	24	40
		dmPFC	L	5.13	0	62	0						4.64	−36	26	36
				4.33	−2	54	6				IFG	L	4.54	−36	28	28
		ACC		4.18	0	36	8				SFG	L	4.43	−22	48	22
1	7	Orbitofrontal cortex	R	6.04	28	34	−10		3	218	Insula	L	7.95	−34	18	−4
				5.99	32	34	−12		2	106		R	6.8	34	22	2
													6.7	30	24	−4
									1	74	Cerebellum	R	7.39	32	−58	−30

Coordinates are given at 3 mm^3^ resolution in MNI space, and max values present peak *t*-value from non-parametric permutation inference. MTG means middle temporal gyrus; v/dmPFC, ventro/dorso mPFC; MCC, midcingulate cortex; I/M/SFG, inferior/middle/superior frontal gyrus; L/R, left/right.

**Table 2:** Clusters of iBBDs expressing the goal-by-role interaction

Builder (COO > COM) > Other (COO > COM)									Other (COO > COM) > Builder (COO > COM)							
Cluster	Voxels	Label		Max	x	y	z		Cluster	Voxels	Label		Max	x	y	z
2	27005	Precuneus	L	9.04	−12	−62	62		3	11776	Calcarine	L	7.96	−10	−86	2
		SFG	L	8.36	−28	−2	66						7.54	−22	−48	4
				8.25	−24	−6	66					R	7.83	24	−50	6
			R	8.18	26	0	62				Cuneus	L	7.56	−2	−86	28
		SPL	L	8.33	−16	−68	56						7.52	−6	−88	24
											Lingual	R	7.52	12	−70	0
		MFG	L	8.21	−26	−2	56		2	1337	MTG	L	5.58	−64	−32	4
1	4733	Cerebellum	L	7.12	−38	−44	−46						5.47	−52	−26	0
				6.03	−24	−34	−42						5.28	−66	−30	0
				5.85	−36	−50	−30						5.14	−60	−38	8
				5.84	−12	−52	−48						5.12	−58	−12	−6
			R	6.56	14	−54	−50				STG	L	4.91	−54	−10	−8
				6.34	30	−40	−46		1	217	Frontal med orbital	L	6.18	−4	50	−4
											ACC	L	6.17	−2	44	8
													5.77	0	40	14

Coordinates are given at 3 mm^3^ resolution in MNI space, and max values present peak *t*-value from non-parametric permutation inference. SPL means superior parietal lobule; STG, superior temporal gyrus; L/R, left/right.

**Table 3:** Clusters of iBBDs expressing the main effect of Interaction (‘left’) and the interactive goal-by-interaction effect (‘right’).

CN > TB									TB (COO > COM) > CN (COO > COM)							
Cluster	Voxels	Label		Max	x	y	z		Cluster	Voxels	Label		Max	x	y	z
4	36 386	STG	R	7.35	58	−44	18		3	14 191	SMA	L	6.37	−8	2	48
		MTG	R	6.96	54	−52	12					R	5.59	10	2	48
				6.55	46	−64	6				Supramarginal gyrus	L	6.09	−58	−24	30
		Precentral	L	6.77	−30	−18	70				Precentral gyrus	L	5.5	−28	−12	54
				6.5	−38	−18	68				SPL	L	5.42	−20	−54	62
		Cerebellum	L	6.76	−14	−76	−36				Postcentral gyrus	R	5.36	56	−22	32
									2	8060	MOG	L	5.8	−40	−70	12
3	335	Thalamus	L	5	−16	−12	12				Vermis		5.67	−2	−62	−8
				4.38	−8	−16	−2						5.18	4	−64	−12
			R	4.46	8	−22	0						5.05	2	−66	−18
		STG	R	5.05	46	−16	−8				MTG	R	4.87	42	−68	4
				4.02	52	−2	−14				Cerebellum	L	4.85	−8	−72	−42
		Insula	R	4.15	40	−10	−6		1	3	Postcentral	R	2.94	36	−32	72
2	275	Temporal pole	R	3.9	54	4	−16									
		Thalamus	R	4.38	16	−8	14									
				3.96	8	−8	4									
				3.88	18	−22	14									
1	120			3.87	12	−12	10									

Coordinates are given at 3 mm^3^ resolution in MNI space, and max values present peak *t*-value from non-parametric permutation inference. MOG means middle occipital gyrus.

#### Role

Consistent with the behavioral data, we observed no differences when contrasting iBBDs brain responses between roles of Builders and Others.

#### Goal structure

Brain responses that represented iBBDs differentiated between cooperative and competitive exchanges. Greater brain responses to the behavior of a co-player were observed in the cooperation compared with the competition condition throughout the bilateral orbitofrontal cortices, mPFC and ACC, putamina and pallida, precunei [extending into the posterior cingulate cortex (PCC)], frontoparietal rolandic opercula, temporal cortices and hippocampi (extending into the amygdalae). In the reverse contrast, we observed differential iBBDs throughout bilateral pre-SMA, triangularis of the inferior frontal cortex and anterior insulae.

#### Interaction structure

Brain responses reflecting iBBDs were greater in the CN relative to the TB condition in the bilateral precentral gyri, temporoparietal cortices and thalami; and the right anterior insula and bilateral superior temporal sulci (STS). No differential expressions of iBBDs were revealed in the reverse contrast.

#### Role-by-goal

Builders exhibited greater differential iBBDs compared with Others in the cooperation relative to the competition condition throughout the frontal and parietal cortices and the cuneus. In Others, however, greater reactive brain responses during the cooperation condition were observed in the mPFC and left ACC, cunei, calcarine cortices and the lingual gyri.

#### Role-by-interaction

In line with the pattern of behavioral data, no differences were observed in interpersonal BBD when contrasting player roles in each level of interaction structure.

#### Goal-by-interaction

We observed stronger iBBDs during competitive exchanges under the CN but not the TB condition; specifically, this was exhibited within the SMA, bilateral precentral and postcentral gyri, supramarginal gyri and occipital cortices. No brain responses expressed this contrast, or the reverse, more in the TB compared with the CN condition.

#### Role-by-goal-by-interaction

As with the behavioral data, the three-way interaction between role, interaction and goal structure revealed no differential iBBDs after thresholding with non-parametric permutations.

## Discussion

Using a dual-fMRI protocol, this study investigated whether discrete dimensions of dyadic social exchange elicit dissociable patterns of interdependency between the behavior of one interactant and the resultant brain responses of another, that is, iBBDs. To this end, we adapted the PG (Decety *et al.*, 2004) to be an experimental paradigm for two-person hyperscanning capable of delineating between different interaction dimensions. Building on Liu and Pelowski (2014)’s framework of social interaction, this is the first research to dissociate between more than one dimension of dyadic exchange and to examine interpersonal brain and behavioral processes between both interactants during CN and TB exchanges. By modeling the brain responses in each interactant as neural reactions to their partner’s behavior, we able to measure iBBDs online, that is, interpersonal brain processes that emerge between two individuals whose active participation combines to give rise to a dynamic, non-linear, real-world social exchange. Our results reveal that specific patterns of player behavior under each dimension were mirrored by discrete patterns of iBBDs.

It might be argued that the interpersonal brain-behavior relationships we have observed in the present study could have been investigated in a simpler, more classic neuroimaging protocol, whereby the brain of one individual was scanned while they played with another person who was not scanned. While this would capture brain signals that are modulated online by the behavior of an interaction partner, it would present an incomplete picture that considers iBBDs to be a unidirectional process; we would not know how the brain of the other player is modulated by the resultant reciprocal actions. Naturalistic dyadic exchanges are defined by the active participation of both interactants; both competitive and cooperative interactions emerge through a bidirectional to and fro of mutually contingent behavioral exchanges that communicate intentional states, giving rise to unique non-linear dynamics that are created by the two players ‘together’. As such, iBBDs reflects brain processes that are simultaneously both a cause and an effect of an interaction partner’s prior and present behavior, and will emerge in a unique fashion during each exchange. By scanning both interactants’ brains simultaneously, we have measured iBBDs as a bidirectional process of ‘mutual’ dependency as it unfolds online over the course of unique social interactions, thereby capturing the shared intentionality between players (Schilbach *et al.*, 2013). Furthermore, the conditions under which participants interacted with one another were highly similar; both interactants knew that their co-player was in a similar context, allowing for a more ecologically valid context (Schilbach *et al.*, 2010).

Beginning with goal structure, players achieved greater success in the cooperative relative to the competition condition. This was reflected in the brain and in a manner consistent with our hypothesis; reactive brain responses were greater during cooperative than competitive rounds in neural systems implicated in reward processing, namely, the putamen and ventral pallidum (Haber and Knutson, 2010). This converges with previous studies in which cooperative tasks are reported to engage the left caudate and putamen (Krill and Platek, 2012). Cooperation is discussed widely in various evolutionary settings, and is generally considered beneficial to individuals (Rilling *et al.*, 2002; Kurzban *et al.*, 2015; Tomasello and Vaish, 2013). iBBDs exhibited within the basal ganglia might therefore represent the reward experienced during such cooperative dyadic exchange (Haber and Knutson, 2010). This interpretation is in line with the results of Schilbach et al. (2010), who used interactive task to compare pleasantness ratings and brain responses during joint-attention task. These authors report that higher ratings of pleasantness were accompanied by stronger engagement of the ventral striatum, associating subjective experiences with neural systems implicated in reward processing. The second set of reactive brain responses constituted brain areas linked repeatedly to socio-cognitive and -emotional processes; specifically, the ACC and mPFC and the amygdalae (Bickart *et al.*, 2014; Twining *et al.*, 2017). A previous study has also reported the involvement of the ACC during cooperative tasks (Chaminade *et al.*, 2012). Interestingly, the ACC is engaged consistently in tasks that require performance monitoring and adaptive behavior during changing environmental demands and evaluating the decisions of others during social interactions (e.g. Apps *et al.*, 2013). This has led to the proposal that the ACC processes reward in an ‘other-oriented’ reference frame, which can be used to estimate the motivation and, in turn, predict the behavior of others (Apps *et al.*, 2016). This pattern of iBBDs also included the PCC, which converges with the pattern of activations observed by Decety *et al.* (2004) during cooperative rounds of the PG. Strong brain responses to an interaction partner’s behavior in this region during our interactive task suggests that it is involved in the adaptation of our own behavior in response to the inferred intentions of our interaction partner(s). Contrary to our prediction, then, this finding might indicate that individuals attempt to infer the intentional state of their interaction partner more during cooperative than competitive exchanges.

iBBDs elicited during the competitive condition was observed throughout brain areas involved predominantly in movement planning and attention processes: pre-SMA, inferior frontal cortex and anterior insula. The previous research has shown that the pre-SMA is activated reliably during tasks that require response inhibition or switching between stimulus-response rules (Nachev *et al.*, 2008). This may reflect the need for players to respond more adaptively during competitive exchanges, changing their plans in response to their co-player’s behavior. The reactivity of the inferior frontal gyrus may represent the functioning of neural mirroring systems implicated in action understanding (Rizzolatti and Craighero, 2004), which would support motor planning performed by the pre-SMA. The response of the anterior insula during competitive exchanges is also consistent with the previous research (Takahashi *et al.*, 2015). Given the well-documented role of this brain area in subjective feelings states (Walter, 2012; Morelli *et al.*, 2014; Hari *et al.*, 2015), this focus of neural reactivity might reflect affective reactions when monitoring and adapting to another’s behavior.

An important novel aspect of this study is the focus not only on goal but also on interaction structure. To our knowledge, all existing hyperscanning experiments have employed interactive tasks that afford ‘either’ TB (e.g. Tomlin *et al.*, 2006; Babiloni *et al.*, 2007) or concurrent (e.g. Tognoli *et al.*, 2007; Lindenberger *et al.*, 2009; Cui *et al.*, 2012) exchanges, but our modified PG enabled us to compare these two types of interaction structure directly. Our data demonstrate important differences between these types of exchange; first, we recorded a higher rate of successful placements during CN compared with TB. Furthermore, we observed a parallel pattern of stronger iBBDs during CN relative to TB exchanges, particularly in the right STS and temporoparietal cortices. Since these brain regions are associated frequently with mentalizing processes (Frith and Frith, 2006; Van Overwalle, 2009; Walter, 2012; Carlson *et al.*, 2013), we suggest this reflects the greater need for individuals to infer their opponent’s intentions in real time during CN compared with sequential interactions.

Second, while the success rate was comparable on CN and TB exchanges in the cooperative condition, players were significantly more successful in CN rounds of the competition condition. Congruently, increased iBBDs expressed during competitive compared with cooperative rounds was greater under the CN relative to the TB condition. This was exhibited within the SMA, bilateral precentral and postcentral gyri—brain areas linked strongly to movement planning. One possible explanation is that competitive exchanges afforded multiple strategies; players may have spent more time evaluating the playing space and second-guessing their opponent’s upcoming move in the TB condition rather than reacting dynamically to their opponent. On CN rounds of the competition condition, however, in which the quicker player often made more successful token placements, there was less time for such strategic planning. Conversely, under the cooperation condition there is no such plurality of strategies to consider; both players work toward the same shared goal in both CN and turn-taking exchanges.

Although we observed no differences when comparing Builders and Others directly, a role-by-goal interaction revealed different patterns of brain reactivity evoked in each role; while, Builders achieved less successful placements than Others in the cooperation condition, the opposite effect was present under the competition condition. This likely reflects fundamental differences in the nature of the task for Helpers and Hinderers; a single strategy for success was illustrated explicitly to Helpers, since only three token placements enabled the Builder to form the target pattern. In contrast, a number of implicit strategies were available to Hinderers in their impedance of the Builder; they could hinder actively through obstructive placements, for instance, or passively through no placements at all. This is an important aspect of our interactive paradigm, and of social exchanges more generally; while both interactants might pursue the same goal, different social contexts may afford different strategies for each individual. We observed a stronger iBBDs during cooperative exchanges in the Builder compared with the Other throughout frontal and parietal brain regions and the precuneus. The precuneus is thought to be involved in self-referential processes and self-other distinction (Cabanis *et al.*, 2013; Reniers *et al.*, 2014), which suggests the recruitment of these processes when one individual (the Builder) must infer the cooperative intentions behind the actions of another (the Other). On the other hand, iBBDs in the Other during cooperative exchanges were expressed in other brain areas associated with socio-cognitive processes, such as the mPFC and ACC (Völlm *et al.*, 2006; Reniers *et al.*, 2014).

Our findings illustrate the need for social neuroscience research to operationalize carefully the specific dimension(s) of social interaction under investigation. In doing so, hyperscanning permits a characterization of the specific dimensions along which clinical disorders exhibit dysfunctional social behavior, and the identification of underlying inter-brain neuromarkers. To develop our results further, future studies should investigate how these dissociable, role-specific patterns of iBBDs emerge spontaneously during naturalistic exchanges. One way to achieve this is to modify our paradigm by removing the fixed task structure. In many interactive paradigms used for hyperscanning studies, such as those employing economic games (e.g. ultimatum game; Shaw *et al.*^2018^; ^2019^), the asymmetry of player roles is enforced by the very nature of the task. In others, however, such asymmetry is allowed to emerge spontaneously; in the synchronized finger-tapping task used by Konvalinka *et al.* (2014), for example, pairs of participants either mutually adjusted to each other or followed a computer metronome. These authors report a spontaneous emergence of leader–follower relationship, which was accompanied by differential brain responses between players. We instructed interactants about the type of exchange they should perform at any one time, but real interactions are often characterized by a degree of uncertainty about how the other person is going to behave, for example, whether they will decide to cooperate or not. Our interactive task could be adjusted such that players are free to choose their role on a given round, affording an interactive context that resembles real-world dyadic exchange even more closely.

## Supplementary Material

Supplementary Data
