# Supplementary material for: Dissecting social interaction: dual-fMRI reveals patterns of interpersonal brain-behavior relationships that dissociate among dimensions of social exchange
Source: Soc Cogn Affect Neurosci. 2019 Jan 15;14(2):225–35. doi: 10.1093/scan/nsz004 (PMC6374606; doi:10.1093/scan/nsz004)
Supplement: Supplementary Data [file scan-18-244-file009-nsz004_supp.docx]

**General Linear Modelling**

***Main effect of Role***

[(COO_Builder_∩COM_Builder_)>CTL_Builder_] *vs* [(COO_Other_∩COM_Other_)>CTL_Builder_]

***Main effect of Goal***

[(COO_Builder_>CTL_Builder_)∩(COO_Other_>CTL_Builder_)] *vs* [(COM_Builder_>CTL_Builder_)∩(COM_Other_>CTL_Builder_)]

***Main effect of Interaction***

([(COO_Builder_^TB^∩COM_Builder_^TB^)>CTL_Builder_^TB^]∩[(COO_Other_^TB^∩COM_Other_^TB^)>CTL_Builder_^TB^]) *vs*

([(COO_Builder_^CN^∩COM_Builder_^CN^)>CTL_Builder_^CN^]∩[(COO_Other_^CN^∩COM_Other_^CN^)>CTL_Builder_^CN^])

***Role-by-Goal***

[(COO_Builder_>CTL_Builder_)>(COM_Builder_>CTL_Builder_] *vs* [(COO_Other_>CTL_Builder_)>(COM_Other_>CTL_Builder_)]

***Role-by-Interaction***

([(COO_Builder_^TB^∩COM_Builder_^TB^)>CTL_Builder_^TB^]>[(COO_Builder_^CN^∩COM_Builder_^CN^)>CTL_Builder_^CN^]) *vs*

([(COO_Other_^TB^∩COM_Other_^TB^)>CTL_Builder_^TB^]>[(COO_Other_^CN^∩COM_Other_^CN^)>CTL_Builder_^CN^])

***Goal-by-Interaction***

([(COO_Builder_^TB^>CTL_Builder_^TB^)∩(COO_Other_^TB^>CTL_Builder_^TB^)]>[(COO_Builder_^CN^> CTL_Builder_^CN^)∩(COO_Other_^CN^>CTL_Builder_^CN^)]) *vs*

([(COM_Builder_^TB^>CTL_Builder_^TB^)∩(COM_Other_^TB^>CTL_Builder_^TB^)]>[(COM_Builder_^CN^>CTL_Builder_^CN^)∩(COM_Other_^CN^>CTL_Builder_^CN^)])

***Role-by-Goal-by-Interaction***

[(COO_Builder_^TB^>COM_Builder_^TB^)>(COO_Other_^TB^>COM_Other_^TB^)] *vs* [(COO_Builder_^CN^>COM_Builder_^CN^)>(COO_Other_^CN^>COM_Other_^CN^)
